# Supplementary material for: Ethylene Promotes Expression of the Appressorium- and Pathogenicity-Related Genes via GPCR- and MAPK-Dependent Manners in Colletotrichum gloeosporioides
Source: J Fungi (Basel). 2022 May 26;8(6):570. doi: 10.3390/jof8060570 (PMC9224669; doi:10.3390/jof8060570)
Supplement: Supplementary file 1 [file jof-08-00570-s001.zip › manuscript supporting information.pdf]

Supporting Information

Supplemental Figure S1. Statistics of red fluorescence area in FDA-PI dual fluorescence staining.

Dual-fluorescence staining with FDA targeting living cells (bright green) and PI targeting dead cells (red fluorescence), ethylene can significantly promote the red fluorescence area in FDA-PI dual fluorescence staining at 24hpi.

Supplemental Figure S2. qRT-PCR verification of up-regulated differential genes. Bar graph showing the results of qRT-PCR validation of the ET-regulated genes; Visualization of RNA-Seq reads coverage across the *C. gloeosporioides* gene analyzed by IGV software, the orange curves indicate read coverage (log scale), for the six samples, the blue line indicated the gene annotation region, the red lines indicate regions amplified by qRT-PCR primers. Information on ET-inducible genes is provided in Supplementary Table3.

Supplemental Figure S3. qRT-PCR verification of the up-regulated differential genes. Bar graph showing the results of qRT-PCR validation of ET-inducible genes; Visualization of RNA-Seq coverage across the *C. gloeosporioides* gene by IGV software, the orange curves indicate read coverage (log scale) samples, the blue line indicated the gene annotation region, the red lines indicate regions amplified by qRT-PCR primers. Information on ET-inducible genes is provided in Supplementary Table3.

Supplemental Figure S4. Annotation information of genes enriched in extracellular region (GO: 0005576).

(a), ET treatment can significantly promote the expression of the genes encoding for polysaccharide deacetylase, cutinase, multicopper oxidase, cerato-platanin protein and other extracellular hydrolases; (b), ET treatment can significantly promote transcript levels of the genes encoding for cell wall proteins, appressorial structure proteins, glycolipid anchored surface protein, and other extracellular structure components.

Supplemental Table S1. Oligonucleotide primers used for target amplification in this study.

Supplemental Table S2. Principal components analysis.

Supplemental Table S3. Correlation analysis of QPCR and RNAseq.

Supplemental Table S4. GO terms of fungal transcripts that are significantly differentially expressed by ethylene treatment. GOID, GO terms, Class, Pvalue and GeneCount are show.

Supplemental Table S5. Colletotrichum gloeosporioides gene expression profile during ethylene treatment 4hpi. GeneID, log2FC(ET/CK), Pvalue, FPKM_ET, FPKM_CK, Description and NR_name are show.

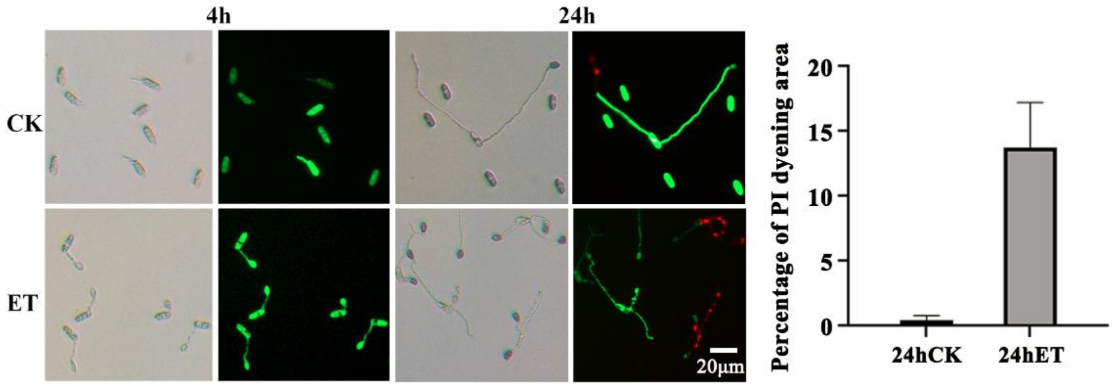

Supplemental Figure S1. Statistics of red fluorescence area in FDA-PI dual fluorescence staining. Dual-fluorescence staining with FDA targeting living cells (bright green) and PI targeting dead cells (red fluorescence), ethylene can significantly promote the red fluorescence area in FDA-PI dual fluorescence staining at 24hpi.

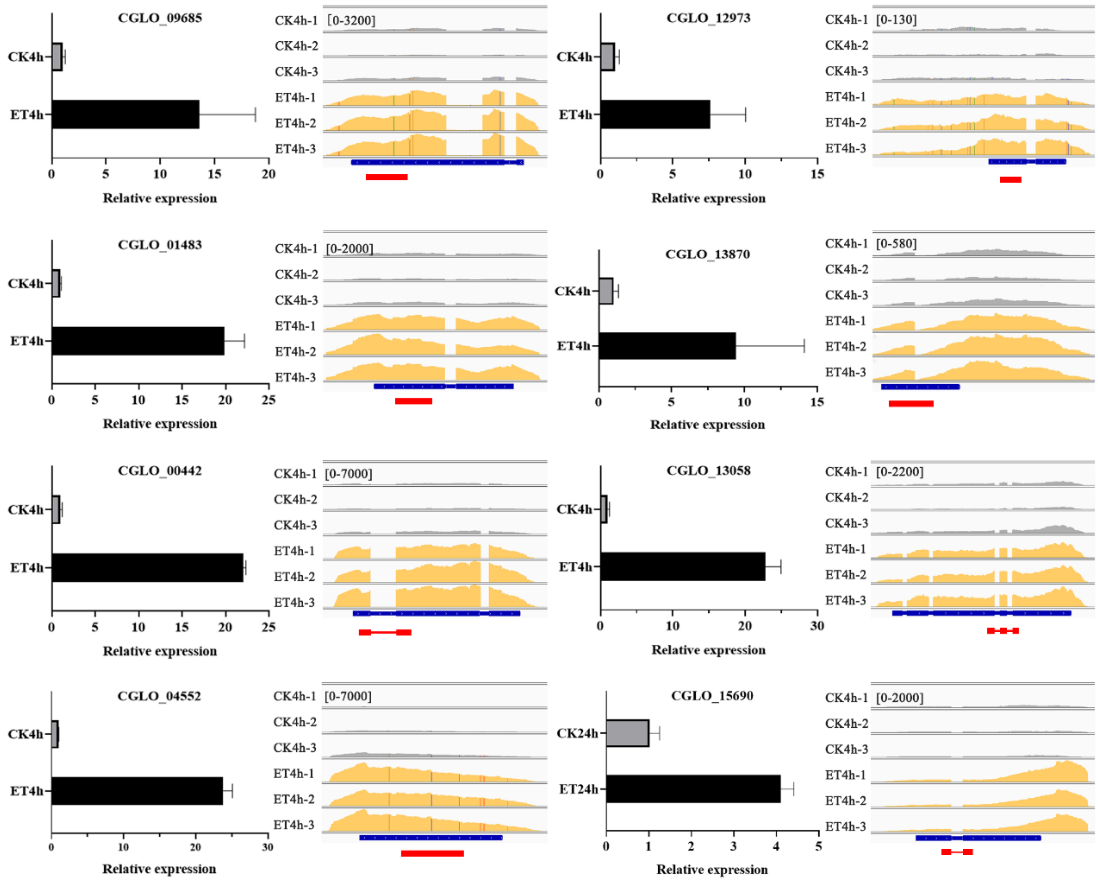

Supplemental Figure S2. qRT-PCR verification of up-regulated differential genes. Bar graph showing the results of qRT-PCR validation of the ET-regulated genes; Visualization of RNA-Seq reads coverage across the C. gloeosporioides gene analyzed by IGV software, the orange curves indicate read coverage (log scale), for the six samples, the blue line indicated the gene annotation region, the red lines indicate regions amplified by qRT-PCR primers. Information on ET-inducible genes is provided in Supplementary Table3.

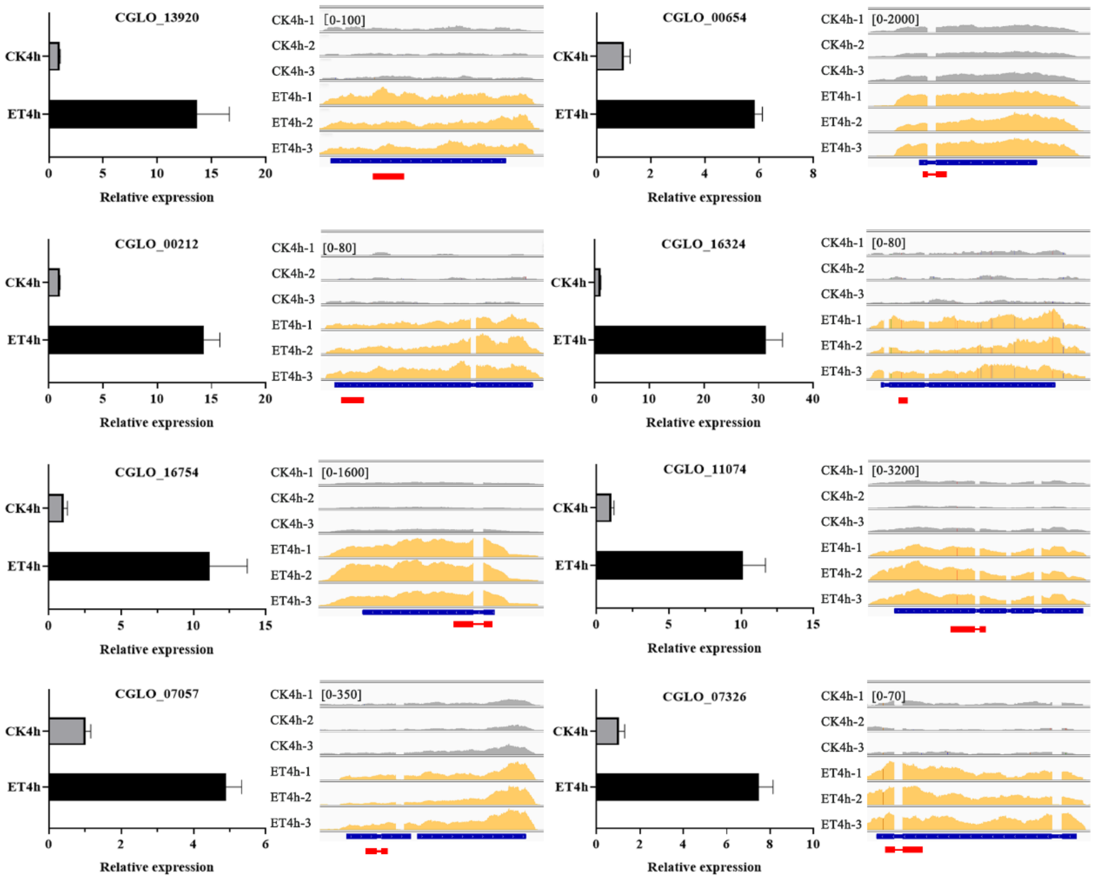

Supplemental Figure S3. qRT-PCR verification of the up-regulated differential genes. Bar graph showing the results of qRT-PCR validation of ET-inducible genes; Visualization of RNA-Seq coverage across the C. gloeosporioides gene by IGV software, the orange curves indicate read coverage (log scale) samples, the blue line indicated the gene annotation region, the red lines indicate regions amplified by qRT-PCR primers. Information on ET-inducible genes is provided in Supplementary Table3.

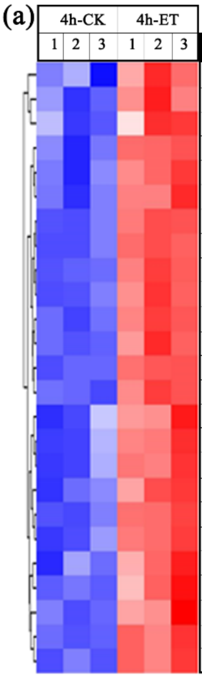

Gene ID	logFC	NR_tophit_description
CGLO_17822	2.89	metalloprotease
CGLO_17052	2.85	L-asparaginase
CGLO_08577	2.62	trypsin
CGLO_17438	2.75	glycosyl hydrolase family 61
CGLO_14874	2.45	calcineurin phosphoesterase
CGLO_15453	2.33	glycosyl hydrolase family 16
CGLO_02789	4.34	catalase
CGLO_16754	2.69	polysaccharide deacetylase
CGLO_01304	3.44	carboxylesterase
CGLO_07214	3.27	serine carboxypeptidase
CGLO_10319	3.78	ribonuclease
CGLO_02394	3.75	multicopper oxidase
CGLO_18083	3.14	glycosyl hydrolase family 92
CGLO_05863	2.85	S1/P1 Nuclease
CGLO_06095	2.40	multicopper oxidase
CGLO_17306	2.85	galactose oxidase
CGLO_12977	2.47	multicopper oxidase
CGLO_13594	2.42	carboxylesterase
CGLO_05657	2.76	alpha amylase
CGLO_06935	2.89	glycosyl hydrolase family 16
CGLO_00212	2.54	polysaccharide deacetylase
CGLO_17126	2.22	cutinase
CGLO_12973	2.26	cerato-platanin
CGLO_03994	3.39	galactose oxidase
CGLO_16353	2.21	glycosyl hydrolase family 76

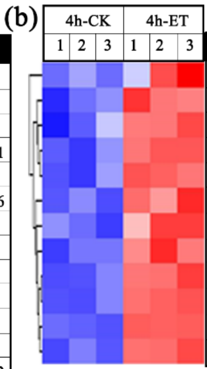

Gene ID	logFC	NR_tophit_description
CGLO_01335	2.95	RAM pathway protein
CGLO_03238	3.75	WSC domain protein
CGLO_04552	2.85	cell wall protein PhiA
CGLO_14273	3.39	FAD binding domain protein
CGLO_02291	2.74	FAD binding domain protein
CGLO_05942	2.59	peroxiredoxin
CGLO_03525	2.82	major facilitator transporter
CGLO_01827	3.63	FAD binding domain protein
CGLO_01483	2.63	CAP22 protein
CGLO_16726	2.59	WSC domain protein
CGLO_05804	4.22	WSC domain protein
CGLO_16605	2.12	glycolipid anchored protein

Supplemental Figure S4. Annotation information of genes enriched in extracellular region (GO: 0005576). (a), ET treatment can significantly promote the expression of the genes encoding for polysaccharide deacetylase, cutinase, multicopper oxidase, cerato-platanin protein and other extracellular hydrolases; (b), ET treatment can significantly promote transcript levels of the genes encoding for cell wall proteins, appressorial structure proteins, glycolipid anchored surface protein, and other extracellular structure components.

Supplemental Table S1 Oligonucleotide primers used for target amplification in this study

Primer	Nucleotide sequence (5'-3')	Target of amplification
**Gene expression level detection related primers**		
actin-QF	GGTCTTTCGAAGAGAAGCCCAG	Primers used for amplification of the actin gene in quantitative real-time PCR
actin-QR	GAGGAAGATGTAGTCGAGCAGGTG	
CGLO_00442-QF	CCTCCGAGTCTAAGGGTTCCAAG	Primers used for amplification of the CGLO_00442 gene in quantitative real-time PCR
CGLO_00442-QR	GTTGGCGTAGTTGACGATGACC	
CGLO_04552-QF	CCAAGTCCTCCATCTTCCTCCAG	Primers used for amplification of the CGLO_04552 gene in quantitative real-time PCR
CGLO_04552-QR	CGTAGGCGTCGATGGTCCAG	
CGLO_13870-QF	GAGGAGCTTGCAGCCCTCGAG	Primers used for amplification of the CGLO_13870 gene in quantitative real-time PCR
CGLO_13870-QR	CGGGTGATGAAGTTACGGATCTCG	
CGLO_11074-QF	CACTGGACTCAACCTGGCTGAAG	Primers used for amplification of the CGLO_11074 gene in quantitative real-time PCR
CGLO_11074-QR	CCATCACATCCAAGATCGAGATGGC	
CGLO_12973-QF	TCTCCGTCTCCTTCGACACCGG	Primers used for amplification of the CGLO_12973 gene in quantitative real-time PCR
CGLO_12973-QR	GGAGTTCCAGCCGGCAATGg	
CGLO_09685-QF	GCTGGCAACATCACCTTCGATG	Primers used for amplification of the CGLO_09685 gene in quantitative real-time PCR
CGLO_09685-QR	GCTGGCAACATCACCTTCGATG	
CGLO_01483-QF	CCTTGGCAACATCTGCACTGTC	Primers used for amplification of the CGLO_01483 gene in quantitative real-time PCR
CGLO_01483-QR	GATGGAGTTGATACCCTCAAGGC	
CGLO_08208-QF	CGTCGCGACCGAACTATTCAACC	Primers used for amplification of the CGLO_08208 gene in quantitative real-time PCR
CGLO_08208-QR	CAACGGTCGCTTCGATGATCCAATC	

CGLO_08904-QF	CCTACCGTTCGCAAGCTAGCG	Primers used for amplification of the CGLO_08904 gene in quantitative real-time PCR
CGLO_08904-QR	CGGTCCTTGGCTGACAGGTTC	
CGLO_13920-QF	CACCTCGCAAGATCCACCACC	Primers used for amplification of the CGLO_13920 gene in quantitative real-time PCR
CGLO_13920-QR	GGCACCATAGTTGACGGTGGTC	
CGLO_00212-QF	GACTGTTGCTGCCACTCTCC	Primers used for amplification of the CGLO_00212 gene in quantitative real-time PCR
CGLO_00212-QR	GCCAGGACGAGAAGGAACTCC	
CGLO_16754-QF	CACTTCCTCTCTCCTGAGCGC	Primers used for amplification of the CGLO_16754 gene in quantitative real-time PCR
CGLO_16754-QR	CTGCTTCAGGATGTCGAGAAGC	
CGLO_07057-QF	CGACCTTATCCATTCAGCTGGC	Primers used for amplification of the CGLO_07057 gene in quantitative real-time PCR
CGLO_07057-QR	CGTCGTCATCTTGGATAGGTCG	
CGLO_00654-QF	CTCCAAGATCCTTACCGTGGC	Primers used for amplification of the CGLO_00654 gene in quantitative real-time PCR
CGLO_00654-QR	CCTCGAACTTCACAGAGGTGC	
CGLO_11074-QF	CTGTGCACGATGACAAGGAGC	Primers used for amplification of the CGLO_11074 gene in quantitative real-time PCR
CGLO_11074-QR	CAGATTGGCCAAGAGGACGATG	
CGLO_07326-QF	CTGTGAATGACGAGAACGACCC	Primers used for amplification of the CGLO_07326 gene in quantitative real-time PCR
CGLO_07326-QR	CTTCGCCTCACGTTGTGCTG	
CGLO_03844-QF	CATCCAGGGTGCTCTTACCAACG	Primers used for amplification of the CGLO_03844 gene in quantitative real-time PCR
CGLO_03844-QR	GGCTTCTTGTTGGTCAGGTCGG	
CGLO_15589-QF	GACGCTGGCTCAAGATGAAAGC	Primers used for amplification of the CGLO_15589 gene in quantitative real-time PCR
CGLO_15589-QR	GAGGTTCAGCTGGTGGATTC	
CGLO_13252-QF	GCAGCTGACCAAGACCCTCATC	

CGLO_13252-QR	GGATGTCGGTCTGAGCCTTTGTC	Primers used for amplification of the CGLO_13252 gene in quantitative real-time PCR
CGLO_11351-QF	GCTCACAGTACACGCTTCCC	Primers used for amplification of the CGLO_11351 gene in quantitative real-time PCR
CGLO_11351-QR	CCACCGTGGAAGTTGAGTGC	
CGLO_03249-QF	CGTTGTCAGCGTCGCTGTC	Primers used for amplification of the CGLO_03249 gene in quantitative real-time PCR
CGLO_03249-QR	GCATCGATCTTCTGGAAAGCGC	
CGLO_07611-QF	GCACCAATTCGATCGATCGC	Primers used for amplification of the CGLO_07611 gene in quantitative real-time PCR
CGLO_07611-QR	GACATGGAAATCTGTCACGTTG	
CGLO_01303-QF	CGATCACTTGCTCATTGGCTC	Primers used for amplification of the CGLO_01303 gene in quantitative real-time PCR
CGLO_01303-QR	GGACGATTTGCTCGTTGTCG	
CGLO_02789-QF	CTCCTGCTCCAGGACTTCCAC	Primers used for amplification of the CGLO_02789 gene in quantitative real-time PCR
CGLO_02789-QR	GGTCTTCTTGCCGACCTGGTTG	
CGLO_10460-QF	CATCTCCGCGCTCTATCAGTC	Primers used for amplification of the CGLO_10460 gene in quantitative real-time PCR
CGLO_10460-QR	GAACTCGACCAGATCCGTCC	
CGLO_07307-QF	CTCACAAGTCGACCTACACCG	Primers used for amplification of the CGLO_07307 gene in quantitative real-time PCR
CGLO_07307-QR	GCAATTCTGCTGGTCGTAGACG	
CGLO_05804-QF	GGCCTTATGGCCATGCCTTTG	Primers used for amplification of the CGLO_05804 gene in quantitative real-time PCR
CGLO_05804-QR	CATCTGGGTGCTCATCGAGTC	
**Deletion and identification related primers of cgcap22 mutants**		
P1	TTAAGAAGGAGCCCTTCACCGTGAAACTCGACAGCGGCTTAC	Primers to amplify upstream fragment for generating cgcap22 deletion mutant
P2	gcccgaatcgggaatgcggctctagTTTGAAGGTTGGGGGGTTGTTG	
P3	GGGTCGGCGCGCCCACCCTTGCTGGCATTTGGTTCGTCCCAG	

P4	tgattactaacagatatcaagcttACGCCTTGCCTGGCTGGGTCTTTG	Primers to amplify downstream fragment for generating cgcap22 deletion mutant
P5	CAACAACCCCCCAACCTTCAAActagagccgcattcccgattcgggc	Primers to amplify NAT resistance fragment for generating cgcap22 deletion mutant
P6	CAAAGACCCAGCCAGGCAAGGCGTaagcttgatatctgttagtaatca	
P7	ATGCAGGCCAAGATCGTCGCC	Primers to identify cgcap22 deletion mutant
P8	TTAGAGCATGAAGGCGCCAAGG	
P9	GTGAGTAATTGAGCCACACTG	
P10	CGGTCATCATGACGTCCAAG	
**Deletion and identification related primers of cgcp1 mutants**		
P11	TTAAGAAGGAGCCCTTCACCCGCCTCATCCTTTCGAGAGTCG	Primers to amplify upstream fragment for generating cgcp1 deletion mutant
P12	gcccgaatcgggaatgcggctctagTTTGGCTGTTGTGGTGGGTGG	
P13	GGGTCGGCGCGCCCACCCTTGACGACGATTCCCTCTTCAACT	Primers to amplify downstream fragment for generating cgcp1 deletion mutant
P14	tgattactaacagatatcaagcttATCACCACACGACCTGGGCG	
P15	CCACCCACCACAACAGCCAAActagagccgcattcccgattcgggc	Primers to amplify NAT resistance fragment for generating cgcp1 deletion mutant
P16	CGCCCAGGTCGTGTGGTGATaagcttgatatctgttagtaatca	
P17	ATGCAGTTCTCCAACCTCGTC	Primers to identify cgcp1 deletion mutant
P18	TTACAGACCGCAGGCGCTGAG	
P19	GTAACGTTGCTCGGCTGGATTG	
P20	CCTAACCAGACACAGGAACC	
**Deletion and identification related primers of cgmk1 mutants**		
P21	TTAAGAAGGAGCCCTTCACCGCGTTGGACCGGACCTCCTTCG	Primers to amplify upstream fragment for generating cgmk1 deletion mutant
P22	gcccgaatcgggaatgcggctctagCTTTGGGGTATTTCGAGGTTG	

P23	GGGTCGGCGCGCCCACCCTTGACCAGAGCATAAGGTGGACGAA	Primers to amplify downstream fragment for generating cgmk1 deletion mutant
P24	tgattactaacagatatcaagcttTTCGTCCACCTTATGCTCTGGT	
P25	CATCGTCCTATACCAGCGACCctagagccgcattcccgattcgggc	Primers to amplify NAT resistance fragment for generating cgmk1 deletion mutant
P26	TTCGTCCACCTTATGCTCTGGTGaagcttgatatctgttagtaatca	
P27	ATGTCGCGCGCGAATCCCCC	Primers to identify cgmk1 deletion mutant
P28	TTCGTCCACCTTATGCTCTGGT	
P29	CAACTTCAGACTTGCTGGCC	
P30	TTCGTCCACCTTATGCTCTGGT	
**Deletion and identification related primers of cgst11 mutants**		
P31	TTAAGAAGGAGCCCTTCACCGCGCTGAGTAATTCCTCAGGTAC	Primers to amplify upstream fragment for generating cgst11 deletion mutant
P32	gcccgaatcgggaatgcggctctagCTTTGGGGTATTTCGAGGTTG	
P33	GGGTCGGCGCGCCCACCCTTGAACGTGGCCACGCAGACGATAC	Primers to amplify downstream fragment for generating cgst11 deletion mutant
P34	tgattactaacagatatcaagcttCCACTCGCCGAGCGCAGATCTG	
P35	CAACCTCGAAATACCCCAAAGctagagccgcattcccgattcgggc	Primers to amplify NAT resistance fragment for generating cgst11 deletion mutant
P36	GTATCGTCTGCGTGGCCACGTTaagcttgatatctgttagtaatca	
P37	ATGGCAATGCTAGCCCCCAAG	Primers to identify cgst11 deletion mutant
P38	TTATGTGATGGGCGTAAGGAAG	
P39	GGTCGTAGGTAGACAATGCG	
P40	GATCGATCCATGACTGTTCCTC	
**Deletion and identification related primers of cggpcr3-1 mutants**		
P41	TTAAGAAGGAGCCCTTCACCGCATGGGATCTGCAGATGCAAC	

P42	gcccgaatcgggaatgcggctctagACTCGTTGGTCGGCTTCAATATGG	Primers to amplify upstream fragment for generating cggpcr3-1 deletion mutant
P43	GGGTCGGCGCGCCCACCCTTGTGTGAGTCACCTTGTGGTTC	Primers to amplify downstream fragment for generating cggpcr3-1 deletion mutant
P44	tgattactaacagatatcaagcttCTCGGCTGTTGGTGTATATTG	
P45	CCATATTGAAGCCGACCAACGAGTctagagccgcattcccgattcgggc	Primers to amplify NAT resistance fragment for generating cggpcr3-1 deletion mutant
P46	CAATATACACCAACAGCCGAGaagcttgatatctgttagtaatca	
P47	ATGGGAGACAGCCACGGCGC	Primers to identify cggpcr3-1 deletion mutant
P48	TCATACACTAAACGATCCCCAC	
P49	CGTCTGTTGCTCTCTTTCG	
P50	GACAGCTACCGAGGACAGAG	

Deletion and identification related primers of cggpcr3-2 mutants

P51	TTAAGAAGGAGCCCTTCACCCATGCGACAGTCCAGGCTTG	Primers to amplify upstream fragment for generating cggpcr3-2 deletion mutant
P52	gcccgaatcgggaatgcggctctagCTCGCTGTTCAAAGTGTGTAGATGTG	
P53	GGGTCGGCGCGCCCACCCTTATGAGACGGTGCACAAACCAC	Primers to amplify downstream fragment for generating cggpcr3-2 deletion mutant
P54	tgattactaacagatatcaagcttGATATGCCATTGAAAGGATGAGCGT	
P55	CACATCTACACACTTTGAACAGCGAGctagagccgcattcccgattcgggc	Primers to amplify NAT resistance fragment for generating cggpcr3-2 deletion mutant
P56	ACGCTCATCCTTTCAATGGCATATCaagcttgatatctgttagtaatca	
P57	ATGGATCAACCAGAAGAGAAAGTC	Primers to identify cggpcr3-2 deletion mutant
P58	TCATGACCGCGAGGCCTGGTC	
P59	GGCGTTGGCGATGAATTCACAG	

Deletion and identification related primers of cggpcr3-1/2 mutants

P60	CCACAGCTGCAGTCTAGAGCCTCGCTGTTCAAAGTGTGTAG	Primers to amplify upstream fragment for generating cggpcr3-2 deletion mutant
P61	CTACACACTTTGAACAGCGAGGCTCTAGACTGCAGCTGTGG	Primers to amplify HYG resistance fragment for generating cggpcr3-2 deletion mutant
P62	CATCCTTTCAATGGCATATCCGGGATCCGCTTAGACAA	
P63	TTGTCTAAGCGGATCCCGGATATGCCATTGAAAGGATG	Primers to amplify downstream fragment for generating cggpcr3-2 deletion mutant
**Fluorescent strain mutant construction related primers**		
P64	cgacggccagtgaattcgagctGAGGCAGATTTTTGTGGTCGG	Cg-gfp fluorescent strain related primers
P65	cccgggccttggtaccgagctCATGATTACGAATTCGCCAGCAG	
P66	ccgggccttggtaccgagctGAGGCAGATTTTTGTGGTCGG	PCGLO_03844-gfp fluorescent strain related primers
P67	gacggccagtgaattcgagctGACTTGACATTTGACACGGC	
P68	GAACTTGTTGGACAGGGCCATTGTGATTTGTGTGTTGTGTTG	
P69	CAACACAACACACAAATCACAATGGCCCTGTCCAACAAGTTC	
P70	gacggccagtgaattcgagctCGACCAAGACAAATAGGCTCC	PCGLO_13252-gfp fluorescent strain related primers
P71	GAACTTGTTGGACAGGGCCATTTTGATTCACTATCCAGAGTGAT	
P72	ATCACTCTGGATAGTGAATCAAAATGGCCCTGTCCAACAAGTTC	

Supplemental Table S2 Principal components analysis

	PC1	PC2	PC3	PC4	PC5	PC6	PC7	PC8	PC9	PC10	PC11	PC12
**4hET.1**	0.26366	0.08453	-0.05447	-0.64277	0.05518	0.34045	0.34522	0.24225	-0.06597	0.01736	-0.43553	0.12676
**4hET.2**	0.22780	0.15877	0.31960	-0.19318	0.47952	-0.39280	0.00767	0.20060	-0.48914	-0.04058	0.33168	-0.08940
**4hET.3**	0.15149	0.26194	0.18523	-0.28172	0.38369	0.16602	-0.41499	-0.45349	0.47216	0.12266	0.05710	-0.02964
**4hCK.1**	0.04860	0.52403	0.06614	0.13755	-0.20185	-0.12392	0.47899	-0.51463	-0.19304	0.32567	-0.07388	-0.02385
**4hCK.2**	0.07077	0.47938	0.01889	0.00949	-0.32555	-0.05694	-0.21285	0.59215	0.24595	0.41385	0.15924	-0.04949
**4hCK.3**	0.04214	0.53271	0.03408	0.07704	-0.19679	0.08543	-0.18346	0.00500	-0.08544	-0.77926	-0.08603	0.07543
**24hET.1**	0.45230	-0.15683	0.14622	0.01935	-0.14752	-0.05190	0.47199	0.01184	0.47446	-0.24940	0.45729	0.07260
**24hET.2**	0.46395	-0.16348	0.43557	0.33906	-0.06852	-0.17032	-0.20528	0.02416	-0.01379	0.10309	-0.48554	0.36105
**24hET.3**	0.46946	-0.19648	-0.08358	-0.16104	-0.45340	0.21625	-0.31821	-0.21226	-0.35898	0.06588	0.18245	-0.37808
**24hCK.1**	0.24620	0.11191	-0.40807	0.31089	0.26733	0.42233	-0.03842	0.02169	-0.21169	0.13094	0.29511	0.51270
**24hCK.2**	0.27520	0.08708	-0.16318	0.42981	0.36117	0.15985	0.14281	0.15931	0.11519	-0.04246	-0.26523	-0.64663
**24hCK.3**	0.26444	0.02844	-0.66592	-0.15023	0.02037	-0.62766	-0.10999	-0.09102	0.12711	-0.04801	-0.15246	0.08240

Supplemental Table S3 Correlation analysis of QPCR and RNAseq

ID	fold change	Log2 fold change(qPCR)	Log2 fold change(RNA-seq)	Description
CGLO_00442	22.0353	4.461744633	2.877	THR1
CGLO_04552	23.797115	4.572714777	2.849	Cell wall protein phiA
CGLO_13870	9.395725	3.232004488	1.438	polyketide synthase
CGLO_11074	10.062866	3.330969352	1.76	Manganese transporter SMF1
CGLO_12973	7.6074728	2.92741727	2.2605	cerato-platanin
CGLO_09685	13.595699	3.765078422	2.881	SCD1
CGLO_01483	19.858043	4.311651548	2.626	CAP22 protein
CGLO_08208	0.1740302	-2.522590412	-1.965	FAD binding domain-containing protein
CGLO_08904	0.4268419	-1.228226293	-1.495	hypothetical protein
CGLO_13920	13.64735141	3.770549084	1.801	WD repeat-containing protein
CGLO_00212	14.27042576	3.834956474	2.544	polysaccharide deacetylase
CGLO_16754	11.09566601	3.471924362	2.69	polysaccharide deacetylase
CGLO_07057	4.898853127	2.292444039	1.272	polysaccharide deacetylase
CGLO_00654	5.830917258	2.543722851	1.061	hypothetical protein
CGLO_07326	7.483266722	2.903668197	2.38	hypothetical protein
CGLO_03844	42.15431418	5.397608383	4.449	cell wall protein
CGLO_15589	56.09184781	5.809719205	5.067	DJ-1/PfpI family protein
CGLO_13252	47.49807406	5.569797112	4.176	hypothetical protein
CGLO_11351	32.18476736	5.008306136	4.759	Superoxide dismutase

CGLO_03249	50.8814551	5.669068024	4.603	hypothetical protein
CGLO_07611	35.18392275	5.136844436	4.6	hypothetical protein
CGLO_01303	23.31791181	4.543366692	4.139	hypothetical protein
CGLO_02789	39.11849297	5.289778887	4.339	catalase
CGLO_10460	29.56653044	4.885893052	4.087	hypothetical protein
CGLO_07307	52.26538594	5.707783897	4.364	kelch domain-containing protein
CGLO_05804	26.19961059	4.711473464	4.219	WSC domain-containing protein

Supplemental Table S4 GO terms of fungal transcripts that are significantly differentially expressed by ethylene treatment. GOID, GO terms, Class, Pvalue and GeneCount are show

GOID	GO terms	Class	Pvalue	GeneCount
**GO terms of fungal transcripts that are significantly up-regulated by ethylene treatment 4 hpi**				
GO:0031225	anchored component of membrane	cellular_component	1.81E-06	22
GO:0016209	antioxidant activity	molecular_function	2.89E-06	16
GO:0004601	peroxidase activity	molecular_function	3.13E-06	14
GO:0005576	extracellular region	cellular_component	4.47E-06	143
GO:0016684	oxidoreductase activity, acting on peroxide as acceptor	molecular_function	5.67E-06	14
GO:0019863	IgE binding	molecular_function	4.76E-05	7
GO:0019865	immunoglobulin binding	molecular_function	4.76E-05	7
GO:0009986	cell surface	cellular_component	4.79E-05	33
GO:0031226	intrinsic component of plasma membrane	cellular_component	0.000111816	63
GO:0009277	fungal-type cell wall	cellular_component	0.000147678	17
GO:0005199	structural constituent of cell wall	molecular_function	0.000148177	3
GO:0042438	melanin biosynthetic process	biological_process	0.000167855	10
GO:0017171	serine hydrolase activity	molecular_function	0.000183369	19
GO:0008236	serine-type peptidase activity	molecular_function	0.000184435	16
GO:0005618	cell wall	cellular_component	0.000331261	43
GO:0016811	hydrolase activity, acting on carbon-nitrogen (but not peptide) bonds, in linear amides	molecular_function	0.000421455	16

GO:0016810	hydrolase activity, acting on carbon-nitrogen (but not peptide) bonds	molecular_function	0.000509917	23
GO:0030158	protein xylosyltransferase activity	molecular_function	0.000569293	3
GO:0035252	UDP-xylosyltransferase activity	molecular_function	0.000569293	3
GO:0016722	oxidoreductase activity, oxidizing metal ions	molecular_function	0.000579583	8
**GO terms of fungal transcripts that are significantly down-regulated by ethylene treatment 4 hpi**				
GO:0006950	response to stress	biological_process	1.34E-09	285
GO:0009628	response to abiotic stimulus	biological_process	4.40E-09	177
GO:0051716	cellular response to stimulus	biological_process	5.33E-09	314
GO:0050896	response to stimulus	biological_process	3.21E-08	416
GO:0000731	DNA synthesis involved in DNA repair	biological_process	6.98E-08	24
GO:0080134	regulation of response to stress	biological_process	9.29E-08	94
GO:0033554	cellular response to stress	biological_process	2.39E-07	190
GO:0048583	regulation of response to stimulus	biological_process	2.64E-07	151
GO:0050794	regulation of cellular process	biological_process	3.02E-07	312
GO:0006979	response to oxidative stress	biological_process	3.29E-07	89
GO:0009411	response to UV	biological_process	5.10E-07	34
GO:0006281	DNA repair	biological_process	8.40E-07	71
GO:0035861	site of double-strand break	cellular_component	1.22E-06	21
GO:0090734	site of DNA damage	cellular_component	1.22E-06	21
GO:0044454	nuclear chromosome part	cellular_component	1.59E-06	66

GO:0045003	double-strand break repair via synthesis-dependent strand annealing	biological_process	2.23E-06	11
GO:0034599	cellular response to oxidative stress	biological_process	2.86E-06	57
GO:0044764	multi-organism cellular process	biological_process	3.42E-06	52
GO:0010118	stomatal movement	biological_process	3.68E-06	19
GO:0009314	response to radiation	biological_process	3.91E-06	72
**GO terms of fungal transcripts that are significantly differentially expressed treatment 24 hpi**		**by ethylene**		
GO:0000003	reproduction	biological_process	0	454
GO:0000910	cytokinesis	biological_process	0	104
GO:0003006	developmental process involved in reproduction	biological_process	0	292
GO:0005515	protein binding	molecular_function	0	675
GO:0006139	nucleobase-containing compound metabolic process	biological_process	0	732
GO:0006351	transcription, DNA-templated	biological_process	0	376
GO:0006355	regulation of transcription, DNA-templated	biological_process	0	341
GO:0006464	cellular protein modification process	biological_process	0	406
GO:0006793	phosphorus metabolic process	biological_process	0	479
GO:0006796	phosphate-containing compound metabolic process	biological_process	0	443
GO:0006810	transport	biological_process	0	734
GO:0006996	organelle organization	biological_process	0	592
GO:0007049	cell cycle	biological_process	0	382
GO:0007154	cell communication	biological_process	0	527

GO:0007163	establishment or maintenance of cell polarity	biological_process	0	98
GO:0007165	signal transduction	biological_process	0	426
GO:0007275	multicellular organism development	biological_process	0	579
GO:0008104	protein localization	biological_process	0	454
GO:0009059	macromolecule biosynthetic process	biological_process	0	628
GO:0009889	regulation of biosynthetic process	biological_process	0	487

Supplemental Table S5 Colletotrichum gloeosporioides gene expression profile during ethylene treatment 4hpi. GeneID, log2FC(ET/CK), Pvalue, FPKM_ET, FPKM_CK, Description and NR_name are show

GeneID	log2FC(ETCK)	Pvalue	FPKM_ET	FPKM_CK	Description	NR_name
**ethylene promoted the Hydrophobic surface binding protein A gene expression**						
CGLO_03844	4.449064211	5.42E-38	50.94638767	2.237024333	HsbA	XP_007274298.1
CGLO_13252	4.176470575	3.21E-23	6.537920333	0.267104	HsbA	EQB47582.1
CGLO_00547	4.178398372	8.26E-14	6.042790667	0.239267667	HsbA	EQB59113.1
CGLO_15619	-1.128859111	0.007472265	5.912797333	13.04913	HsbA	EQB45500.1
CGLO_09362	-0.029865411	0.282406067	2.485886	2.539974667	HsbA	EQB51140.1
CGLO_08778	-0.703009099	0.109797136	30.32419967	49.42745233	HsbA	EQB51658.1
**ethylene promoted the cutinase gene expression**						
CGLO_17337	1.217384692	2.19E-20	57.54139967	24.689354	CTF1α	EQB43948.1
CGLO_17126	2.22324472	3.55E-13	4.813507667	0.952272667	cutinase	EQB44152.1
CGLO_00958	1.338829748	2.42E-06	57.43496333	45.391884	CTF1β	EQB58753.1
CGLO_16577	1.282722721	0.000614238	1.611897	0.603622333	cutinase	EQB44664.1
**ethylene promoted the melanin synthesis gene expression**						
CGLO_05047	1.833153951	1.00E-22	103.0350573	28.84489567	PKS	EQB55056.1
CGLO_09685	2.880763367	4.92E-38	234.491516	31.75049	SCD	EQB50834.1
CGLO_10812	2.871037204	2.16E-57	531.6096193	72.578541	T4HR1	EQB49816.1
CGLO_00442	2.877239309	9.23E-37	616.6463823	83.84044633	THR1	XP_007275196.1
CGLO_10813	2.424821821	4.34E-48	11.48703533	2.057884667	CMR1	EQB49817.1
CGLO_06458	1.075768152	4.69E-10	52.160119	24.69316167	laccase genes	XP_007287641.1

CGLO_02601	1.462844269	9.19E-16	91.22101867	33.02918433	laccase genes	EQB57292.1
CGLO_06095	2.402810583	4.97E-17	15.76718267	2.900418	laccase genes	EQB54113.1
CGLO_12977	2.473574459	2.10E-31	99.16951767	17.772932	laccase genes	EQB47848.1
CGLO_02394	3.75466207	7.83E-59	32.25855633	2.297304667	laccase genes	EQB57469.1
CGLO_04100	1.906031748	2.07E-05	2.205543333	0.515177667	laccase genes	EQB55926.1
**ethylene promoted the chitin deacetylases gene expression**						
CGLO_05235	-0.668802214	0.422105227	2.143086	3.465951667	Chitin deacetylases	EQB54869.1
CGLO_05233	-0.13878617	0.108898024	6.555568333	7.227637	Chitin deacetylases	XP_007285779.1
CGLO_07057	1.272022303	6.61E-14	26.051288	10.72869733	Chitin deacetylases	EQB53242.1
CGLO_16772	0.43098559	1.44E-06	17.77677767	13.160187	Chitin deacetylases	XP_007284987.1
CGLO_00212	2.544167162	2.70E-16	5.590782	0.875666333	Chitin deacetylases	EQB59400.1
CGLO_16754	2.69043504	5.09E-57	189.017222	29.19742367	Chitin deacetylases	EQB44495.1
CGLO_02140	2.631594479	9.39E-27	30.05877267	4.766590333	Chitin deacetylases	EQB57696.1
